# Risk Factors for Financial Toxicity in Patients With Pancreatic Cancer

**DOI:** 10.1002/cam4.70799

**Published:** 2025-04-18

**Authors:** Jing Wang, Jialu Cui, Xiaoyuan Wang, Zhihua Li, Yang Liu, Baoxin Shi

**Affiliations:** ^1^ Hospice Care Research Center Tianjin Medical University Tianjin China; ^2^ TianJin Medical University Cancer Institute and Hospital Tianjin Medical University Tianjin China

**Keywords:** financial toxicity, pancreatic cancer, psychological distress, quality of life, social support

## Abstract

**Background:**

The increasing costs of cancer treatment impose a tremendous economic burden on patients and their families, adversely impacting their quality of life and patients' outcomes. Financial toxicity (FT), as a concept describing the economic burden on patients, is crucial to comprehend the extent and determinants within specific contexts.

**Objective:**

To understand the current status of FT among Chinese pancreatic cancer (PC) patients, identify risk factors for FT, and summarize the characteristics of high‐risk groups.

**Methods:**

A cross‐sectional study involving 296 PC patients was conducted to investigate their general information, financial toxicity, quality of life, positive and negative affects, and social support. Univariate and multiple linear regression analyses were used to examine the correlation between FT and other variables.

**Results:**

The PC patient's FT score was 54.27 ± 14.50, with 25.7% being forced to change their work status due to the disease diagnosis and 29.4% exhibiting economically related treatment nonadherence behaviors. Factor analysis showed financial toxicity associated with fewer household savings, more total out‐of‐pocket (OOP) costs, treatment nonadherence, unemployment, diminished positive affect, and insufficient social support.

**Conclusions:**

FT was highly prevalent among PC patients and associated with factors such as household savings and total OOP costs. There was a need to identify and manage patients exhibiting high‐risk characteristics and to implement targeted interventions to mitigate their economic burden.

## 1 Introduction

Pancreatic cancer is a common malignancy with poor prognosis and high mortality [1], with a 5‐year survival rate ranging from only 2% to 12% [2]. Moreover, with the growth of the aging population and changes in modern lifestyles, the incidence of PC will continue to rise for a long time, posing a remarkable challenge to the overall health and economy [1, 3]. The progress of science has provided novel therapeutic options, which often have higher clinical and cost‐effectiveness. When the growing number of PC patients receives more expensive interventions, social costs begin to rise, especially when these interventions yield little benefit. At the same time, it needs to be noted that these social costs are being transferred to patients in an unnoticed and unavoidable manner until the patient's consumption ceiling is breached.

PC places a heavy burden on patients and the healthcare system, with the cost of PC care in the US estimated to be $2.7 billion in 2017 [4]. A review of 26 studies examining the PC economic burden in Europe revealed that the average cost for a European PC patient was €40,357 (ranging from €802 to €353,099), with an average cost of €3656 per patient per month [5]. In contrast, colorectal cancer, the third most common cancer in the world, had an average cost that varied between €259 and €36,295 [6]. This is because PC often exhibits a stealthy onset and lacks specific early symptoms, with most patients being diagnosed at an advanced stage, and the highly specialized treatment required for advanced PC further exacerbates the economic burden of the disease. According to the prediction by Draus et al. [7], the total cost of PC patients in Sweden will be between €210 and €225 million in 2030; PC will bring higher economic costs and greater production losses for society. In China, despite basic medical insurance being universal, PC patients still face significant health‐related economic challenges. Patients in China diagnosed with metastatic pancreatic cancer incurred monthly medication expenses ranging from 5446 to 22,497 Chinese yuan (CNY), with total cumulative costs over 5 years reaching between 85,172 and 143,236 CNY [8]. This economic burden stems from the limitation of basic health insurance [9], which only covers drugs, diagnoses, and treatment costs stipulated by the government. Nonetheless, a considerable proportion of anticancer drugs in China, mainly imported ones, remain uninsured [10, 11], resulting in a lower reimbursement ratio for cancer treatment than for other chronic diseases. Cancer patients have to bear more expenses during treatment. In summary, the economic burden on cancer patients and their families in China is immense due to uneven medical insurance coverage, unbalanced medical insurance reimbursement ratios, and high prices of cancer drugs.

The heavy economic burden does not just affect patients' treatment options; it also leads to numerous adverse consequences such as a decline in quality of life, psychological distress, and behavioral changes [12, 13], thereby affecting the treatment outcomes and prognosis [14]. As a concept describing the economic burden of cancer patients, financial toxicity [15] includes both objective economic burden and subjective financial distress, which is a complex phenomenon driven by a variety of demographics, disease and treatment, material burden, and psychological distress. In cancer research, FT correlates with lower quality of life, poorer treatment adherence, and early death [16, 17]. Severe financial distress resulting in bankruptcy has even been proven to be an independent risk factor for death [18]. The escalating expenses of cancer care necessitate urgent investigation and intervention regarding FT as a serious side effect of treatment [19].

Measuring the FT of PC patients by scientific means and identifying the main factors influencing their economic situation not only provides a path to reduce their economic stress but also offers the possibility of alleviating the burden on public health. However, the understanding of the burden of FT in PC patients is limited. Therefore, this study aims to assess the current status of FT among Chinese PC patients, identify the risk factors for FT, summarize the characteristics of high‐risk groups, and provide a reference for developing targeted interventions and policies.

## 2 Methods

### 2.1 Design and Participants

A cross‐sectional survey was conducted from April 2024 to August 2024 at the Tianjin Medical University Cancer Institute and Hospital, recruiting PC patients who were undergoing or had completed treatment using a convenience sampling method. The hospital is a renowned Grade 3A specialized cancer hospital with 2445 beds, 1.89 million annual outpatient visits, and 147,000 annual inpatients [20]. Meanwhile, the Pancreatic Oncology Department of this hospital is the largest pancreatic tumor diagnosis and treatment center in northern China, treating more than 30,000 patients annually, and completing more than 600 various pancreatic surgeries on average each year [21].

The inclusion criteria were as follows: (1) a diagnosis of pancreatic cancer; (2) age of 18 years or older; (3) illness duration exceeding 1 month; (4) conscious, with the ability to communicate and comprehend the survey; (5) and voluntary participation in this study and signed informed consent. The exclusion criteria were as follows: (1) critically ill and unable to bear the research load; (2) not receiving cancer care or participating in any ongoing clinical trial; (3) unaware of their diagnosis; and (4) suffering from severe mental illness or cognitive impairment.

According to Kendall's sample size calculation principle [22], the sample size should be 5–10 times the number of study variables. This study included 30 variables and expanded the sample size to 188–375, considering the presence of invalid questionnaires.

## 3 Measures

### 3.1 General Information

A self‐designed questionnaire was employed to assess the participants' general information, informed by a literature review aligned with the study objectives. Demographic information included sex, age, place of residence, educational level, marital status, living conditions, and number of children. Disease‐related information included number of hospitalizations, duration of illness, clinical stage, presence of lymphatic metastases, presence of distant metastases, type of treatment received, and number of chronic diseases. The number of hospitalizations refers to the cumulative number of all hospitalizations for PC treatment or diagnosis since the patient's confirmed diagnosis of PC. Chronic diseases refer to diseases such as diabetes and hypertension that require long‐term management. Financial information included work status, monthly income, household savings, type of medical insurance, total OOP costs, knowledge of OOP costs, work status before diagnosis, work status after diagnosis, impact of disease on work, presence of economically related treatment nonadherence behaviors, and treatment nonadherence behaviors. The disease‐related work change was designed to assess the impact of the diagnosis on the patient's employment and whether the patient was forced to increase/decrease their income due to the illness, including overtime/part‐time job/delaying retirement age, early retirement age, unemployment/resignation at home, and taking leave/closing the business. Economically related treatment nonadherence behaviors were utilized to assess patients' coping strategies toward FT, measured using multiple‐choice questions based on literature review [23, 24, 25], including interrupting therapy, delaying therapy, reducing the frequency or dose of medication, delaying treatment/consultation, and abandoning or changing the treatment plan for financial reasons. Patients answered these questions with “yes” or “no,” and those who answered “yes” to at least one question were classified as “treatment non‐adherent.”

#### 3.1.1 Patient Reported Outcome for Fighting FInancial Toxicity (PROFFIT)

PROFFIT was used to evaluate participants' FT. PROFFIT was developed and validated by Riva et al. [26, 27], and our team translated and adapted the Chinese version, which has shown satisfactory psychometric properties among cancer patients. The scale consisted of 16 items, 7 of which for measuring patients' FT severity (PROFFIT score, Items 1–7, range 0–100, 100 indicating maximum FT), and the other 9 assessed possible determinants of FT (FT determinants, Items 8–16, range 0–100 for each item, 100 indicating the greatest impact on FT). In FT determinants, the nine possible determinant items were divided into four dimensions: disease treatment costs (FACT1, Items 8 and 9), complementary treatment costs (FACT2, Items 10 and 11), nonmedical costs (FACT3, Items 12 and 13), and medical staff support (FACT4, Items 14–16).

#### 3.1.2 The Functional Assessment of Cancer Therapy–Hepatobiliary Questionnaire (FACT‐Hep)

FACT‐Hep [28] was used to measure the quality of life of subjects. The scale was divided into five dimensions: physical, social/family, emotional, functional, and specific issues well‐being, with a total of 45 items. The scale was scored using a 5‐point Likert scale (0 = “not at all” to 3 = “very much”), with total scores ranging from 0 to 180. Higher scores indicated a greater quality of life. FACT‐Hep has proven valid and reliable and has already been used in a Chinese context [29].

#### 3.1.3 Positive and Negative Impact Scale (PANAS)

PANAS [30], used to assess the psychological well‐being of the participants, was divided into two subscales of positive and negative affect, with a total of 20 items. Each item was rated on a 5‐point Likert scale, and the sum of the scores of each item was the final score; higher scores indicated more intense positive or negative emotions. The psychometric properties of the simplified Chinese PANAS were reported by Huang et al. [31]. In this study, we used the positive and negative affect subscales separately to better observe the differences in the emotional structure of patients.

#### 3.1.4 Social Support Rating Scale (SSRS)

SSRS [32] was used to assess patients' social support, including three dimensions—objective support, subjective support, and social support utilization. The scale consisted of 10 items with scores ranging from 12 to 66. The higher the score, the better the social support. SSRS had excellent psychometric properties, with a Cronbach's *α* coefficient of 0.90.

### 3.2 Data Collection

The study followed the principles of voluntariness, anonymity, and confidentiality. During the survey, a uniform set of instructions was employed to clarify the study's purpose and significance to the patients, inform them about how to fill in the questionnaire, and any points to note. After obtaining their consent and signing an informed consent form, patients were guided to complete the questionnaire independently, which was collected on the spot. The researchers answered any questions the patients had immediately during the filling process. The questionnaire consisted of 106 items and took approximately 20–30 min for participants to complete. A total of 310 questionnaires were distributed, of which 14 were invalid due to missing data or incorrect filling, and 296 valid questionnaires were finally recovered, with a valid response rate of 95.48%.

### 3.3 Statistical Analysis

Statistical analysis was performed using SPSS V.26.0 with a significance level set at 0.05. In descriptive statistics, categorical variables were expressed as frequencies and percentages, whereas continuous variables were expressed as means and standard deviations (SD). A univariate analysis was conducted to compare differences in PROFFIT score and FT‐determinant dimensions across various patient characteristic groups. The independent samples *t*‐test (two‐group comparison) and one‐way ANOVA (multigroup comparison) were employed to compare normally distributed data, while the Mann–Whitney test and the Kruskal–Wallis test were used for comparing abnormally distributed data. If the data followed a normal distribution, Pearson correlation analysis was used to explore the correlation among PROFFIT score, FT determinants, quality of life, positive affect, negative affect, and social support; otherwise, Spearman correlation analysis was used. Univariate and correlation analysis informed the subsequent multiple linear stepwise regression analysis (α_enter_ = 0.05 and α_remove_ = 0.10) to test the influencing factors of PROFFIT score. The collinearity of the selected variables was evaluated by the variance inflation factors (VIF), where a VIF value below 2 signifies the absence of multicollinearity among the independent variables.

## 4 Results

### 4.1 Participant Characteristics

This study investigated 296 patients with PC, and their demographic, disease‐related, and financial data are presented in Table 1. Among them, the median age was 62 years (interquartile range: 54–67), 52.7% were male, 27% of patients had OOP exceeding 100,000 CNY, 31.4% reported a monthly income of 2000 CNY or less, most patients (40.9%) earned between 2000 and 5000 CNY monthly, and only about one‐quarter (27%) indicated that their household savings surpassed 200,000 CNY. 19.6% of patients were diagnosed with Stage IV cancer, the most common form of treatment was surgery (64.1%), and the majority of respondents were retired (43.9%) or unemployed (34.8%).

**TABLE 1:** Samples' general information and univariate analysis of FT (*N* = 296).

Variable	*n*(%)	PROFFIT score^a^ (Mean ± SD)	t/F/Z/K	*p*
**Demographic information**				
Sex				
Male	156 (52.7)	55.69 ± 14.82	1.880	0.060
Female	140 (47.3)	52.68 ± 14.02		
Age (years)				
≤ 55	89 (30.1)	57.29 ± 14.98	7.411**	0.001
56 ~ 65	113 (38.2)	55.67 ± 13.75		
> 65	94 (31.8)	49.71 ± 13.97		
Place of residence				
Cities	149 (50.3)	49.56 ± 14.70	34.470***	< 0.001
Townships	38 (12.8)	55.50 ± 12.79		
Rural	109 (36.8)	60.27 ± 12.44		
Educational level				
Middle school and below	127 (42.9)	58.24 ± 12.86	6.978***	< 0.001
High school/secondary school	120 (40.5)	52.53 ± 14.41		
Junior college	33 (11.1)	48.24 ± 15.95		
Undergraduate and above	16 (5.4)	48.19 ± 16.77		
Marital status				
Single	4 (1.4)	65.25 ± 12.58	1.102	0.349
Married	268 (90.5)	54.29 ± 14.58		
Divorced	6 (2.0)	48.50 ± 14.20		
Widowed	18 (6.1)	53.44 ± 13.57		
Living alone				
Yes	21 (7.1)	54.38 ± 14.75	0.037	0.970
No	275 (92.9)	54.26 ± 14.51		
Number of children				
Childless	10 (3.4)	63.30 ± 17.12	7.471***	< 0.001
One	170 (57.4)	51.03 ± 14.57		
Two	92 (31.1)	58.20 ± 13.26		
Three or more	24 (8.1)	58.37 ± 11.64		
**Disease‐related information**				
Numbers of hospitalizations				
≤ 5	208 (70.3)	51.17 ± 13.39	46.269***	< 0.001
6 ~ 15	58 (19.6)	58.02 ± 15.53		
> 15	30 (10.1)	68.50 ± 8.87		
Duration of illness				
< 3 months	127 (42.9)	50.13 ± 12.62	12.418***	< 0.001
3 ~ 6 months	86 (29.1)	52.85 ± 14.54		
6 months~1 year	20 (6.8)	54.35 ± 13.50		
1 ~ 2 years	35 (11.8)	63.40 ± 13.18		
> 2 years	28 (9.5)	65.89 ± 14.41		
Clinical stage				
I	94 (31.8)	48.82 ± 13.36	10.724***	< 0.001
II	93 (31.4)	53.35 ± 14.15		
III	51 (17.2)	60.22 ± 13.53		
IV	58 (19.6)	59.33 ± 14.33		
Lymphatic metastases				
Yes	157 (53.0)	56.46 ± 14.73	2.803**	0.005
No	139 (47.0)	51.78 ± 13.88		
Distant metastases				
Yes	58 (19.6)	59.33 ± 14.33	3.004**	0.003
No	238 (80.4)	53.03 ± 14.31		
Type of treatment received				
Surgery	234 (64.1)	52.87 ± 14.28	25.539***	< 0.001
Radiotherapy	1 (0.3)	38.00		
Chemotherapy	118 (32.3)	60.06 ± 14.17		
Immunotherapy	2 (0.5)	59.50 ± 23.34		
Target therapy	6 (1.6)	64.33 ± 6.68		
Traditional Chinese medicine	4 (1.1)	51.25 ± 21.98		
Number of chronic diseases				
0	148 (50.0)	55.73 ± 13.79	1.295	0.276
1	86 (29.1)	53.67 ± 15.24		
2	46 (15.5)	51.22 ± 15.00		
≥ 3	16 (5.4)	52.69 ± 15.04		
**Financial information**				
Work status				
Full‐time	63 (21.3)	52.95 ± 14.62	47.521***	< 0.001
Retired	130 (43.9)	48.88 ± 14.22		
Unemployed	103 (34.8)	61.86 ± 11.24		
Monthly income (CNY)				
≤ 2000	93 (31.4)	61.68 ± 11.54	55.584***	< 0.001
2001 ~ 5000	121 (40.9)	53.53 ± 12.98		
5001 ~ 8000	46 (15.5)	51.87 ± 14.50		
8001 ~ 11,000	26 (8.8)	43.62 ± 15.83		
> 11,000	10 (3.4)	33.00 ± 10.22		
Household savings (CNY)				
≤ 50,000	4 (1.4)	64.25 ± 9.14	46.416***	< 0.001
50,001 ~ 100,000	12 (4.1)	70.17 ± 11.30		
100,001 ~ 150,000	61 (20.6)	64.05 ± 10.16		
150,001 ~ 200,000	139 (47.0)	56.05 ± 11.26		
> 200,000	80 (27.0)	40.82 ± 12.61		
Type of medical insurance				
Urban workers' medical insurance	181 (61.1)	50.48 ± 14.41	50.894***	< 0.001
Urban resident medical insurance	24 (8.1)	52.42 ± 12.94		
New rural cooperative medical insurance	88 (29.7)	63.06 ± 11.07		
Others	3 (1.0)	39.67 ± 2.89		
Total OOP costs (CNY)				
≤ 20,000	6 (2.0)	45.17 ± 17.11	20.698***	< 0.001
20,001 ~ 50,000	105 (35.5)	47.27 ± 12.45		
50,001 ~ 100,000	105 (35.5)	55.76 ± 13.20		
> 100,000	80 (27.0)	62.18 ± 13.99		
Knowledge of OOP costs				
Very well	180 (60.8)	55.34 ± 14.87	1.090	0.353
Somewhat	95 (32.1)	53.05 ± 14.22		
Hardly at all	20 (6.8)	50.20 ± 12.11		
Not at all	1 (0.3)	57.00		
Impact of disease on work				
No impact	223 (75.3)	52.60 ± 13.97	37.935***	< 0.001
Yes impact, overtime/part‐time job/delaying retirement age	1 (0.3)	67.00		
Yes impact, early retirement age	9 (3.0)	66.67 ± 9.04		
Yes impact, unemployment/resignation at home	18 (6.1)	71.00 ± 8.65		
Yes impact, taking leave/closing the business	45 (15.2)	53.07 ± 14.72		
Treatment nonadherence				
Yes	87 (29.4)	66.32 ± 10.44	9.564***	< 0.001
No	209 (70.6)	49.25 ± 12.93		

*Note:* *, means *p* < 0.05; **, means *p* < 0.01; ***, means *p* < 0.001.

^a^ Range of 0–100, a higher PROFFIT score indicates greater FT.

Among our respondents, 180 (60.8%) reported being “very well” aware of OOP costs, the remainder had limited knowledge of OOP costs, and only one person was “not at all” aware of OOP costs, with knowledge of OOP costs not related to the patient's FT scores (*F* = 1.090, *p* = 0.353). Forty‐five (15.2%) took leave/closed the business due to cancer care, 18 (6.1%) were unemployed/resigned at home after diagnosis, 9 (3%) reached early retirement age after diagnosis, and 1 (0.3%) took a part‐time job to increase income after diagnosis. In summary, approximately one‐quarter of the respondents (*n* = 73) were forced to change their work status due to illness, and FT was more severe in patients who underwent work changes (*K* = 37.935, *p* < 0.001). Among the population with treatment nonadherence, due to financial issues, 38 (12.8%) abandoned or changed the treatment plan, 33 (11.1%) delayed treatment/consultation, 24 (8.1%) delayed therapy, 6 (2%) reduced the frequency or dose of medication, and 6 (2%) interrupted therapy. The results of univariate analysis showed that the presence of treatment nonadherence was significantly associated with patients' FT scores (*Z* = 9.564, *p* < 0.001), but the difference between the groups was not significant among patients with treatment nonadherence (*F* = 1.706, *p* = 0.155).

### 4.2 Participants' Financial Toxicity and Univariate Analysis

The PROFFIT score of PC patients was 54.27 ± 14.50, indicating a relatively severe overall FT [27]. The results of the univariate analysis showed that there were significant differences in FT scores among patients with different ages, places of residence, educational levels, numbers of children, numbers of hospitalizations, durations of illness, clinical stages, types of treatment received, work statuses, monthly incomes, household savings, types of medical insurance, total OOP costs, impacts of disease on work, and presence of lymphatic metastases, distant metastases, and treatment nonadherence (Table 1).

Appendix Table A1 presents the results of univariate analysis of FT‐determinant dimensions. FACT1 score was 59.97 ± 21.02, and it varied in terms of number of hospitalizations, duration of illness, clinical stage, type of treatment received, work status, monthly income, household savings, type of medical insurance, total OOP costs, knowledge of OOP costs, impact of disease on work, and presence of lymphatic metastases, distant metastases, and treatment nonadherence. FACT2 score was 10.25 ± 17.36, and patients across varying educational levels, duration of illness, type of treatment received, monthly income, total OOP costs, and knowledge of OOP costs exhibited distinct FACT2 scores. FACT3 score was 58.73 ± 40.22 and was related to age, place of residence, educational level, number of children, work status, monthly income, household savings, type of medical insurance, impact of disease on work, and presence of treatment nonadherence. FACT4 score was 6.27 ± 11.73, with variations in place of residence, educational level, household savings, and type of medical insurance observed to affect FACT4 score.

### 4.3 Correlation Between Financial Toxicity, Quality of Life, Positive and Negative Affect, and Social Support

Among the 296 participants, the quality of life score was 101.52 ± 15.09, with the FACT‐Hep scores for each dimension detailed in Table 2; the positive affect score was 13.52 ± 2.38, the negative affect was 13.34 ± 2.15, and social support score was 38.27 ± 5.74. Correlation analysis results indicated a negative correlation between quality of life, positive affect, and social support with PROFFIT score, whereas a positive correlation was observed between negative affect and PROFFIT score (Table 2). All dimensions of FACT‐Hep were negatively correlated with PROFFIT score, with emotional and functional well‐being demonstrating the most robust correlation with FT.

**TABLE 2:** Correlation between financial toxicity, quality of life, positive and negative affect, and social support (*N* = 296).

Variable	Mean ± SD	Correlation with financial toxicity	
		*r*	*p*
Quality of life	101.52 ± 15.09	−0.571	< 0.001
Physical well‐being	13.25 ± 3.65	−0.347	< 0.001
Social/family well‐being	18.03 ± 2.45	−0.440	0.001
Emotional well‐being	13.00 ± 3.09	−0.616	< 0.001
Functional well‐being	17.15 ± 2.77	−0.539	< 0.001
Specific issues well‐being	40.10 ± 7.69	−0.395	< 0.001
Positive affect	13.52 ± 2.38	−0.551	< 0.001
Negative affect	13.34 ± 2.15	0.486	< 0.001
Social support	38.27 ± 5.74	−0.458	< 0.001

In the context of FT‐determinant dimensions, FACT1, FACT3, and FACT4 were negatively correlated with quality of life, positive affect, and social support, and positively correlated with negative affect; except for the physical well‐being of FACT‐Hep, FACT2 was not significantly associated with any of the scales (Appendix Table A2).

### 4.4 Multivariate Analysis of FT in PC Patients

The assignment of independent variables is shown in Appendix Table A3, where dummy variables were set for unordered multicategorical variables. Using PROFFIT score as the dependent variable, a multiple linear stepwise regression analysis was conducted with variables that exhibited statistical significance in univariate and correlation analysis as independent variables. Variables possessing clinical significance (number of chronic diseases and knowledge of OOP costs) were also included in the regression model. As shown in Table 3, the model accounts for 70.1% of FT, and the risk factors independently associated with FT included household savings, total OOP costs, positive affect, presence of treatment nonadherence, social support, and work status. All VIFs for the multivariate analysis were below 2. There was no multicollinearity among the selected variables.

**TABLE 3:** Multiple linear regression analysis of FT in PC patients (*N* = 296).

Variables	*B*	SB	B′	*t*	*p*	95% confidence interval
Constant term	96.631	3.857	—	25.053	< 0.001	(89.040, 104.223)
Household savings	−5.037	0.656	−0.303	−7.681	< 0.001	(−6.327, −3.746)
Total OOP costs	5.915	0.614	0.339	9.632	< 0.001	(4.706, 7.124)
Positive affect	−1.637	0.237	−0.269	−6.916	< 0.001	(−2.103, −1.171)
Treatment nonadherence	6.196	1.172	0.195	5.286	< 0.001	(3.889, 8.504)
Social support	−0.454	0.104	−0.179	−4.380	< 0.001	(−0.658, −0.250)
Work status	−4.173	1.016	−0.143	−4.109	< 0.001	(−6.172, −2.174)

*Note: R* = 0.841, *R* ^2^ = 0.707, adjusted *R* ^2^ = 0.701, *F* = 129.330, *p* < 0.001.

### 4.5 PROFFIT Score and FT Determinants

The correlation of PROFFIT score with FACT1, FACT3, and FACT4 was significant, whereas the correlation with FACT2 did not reach statistical significance. Further analysis revealed that the PROFFIT score exhibited a weaker correlation with Items 10, 11, and 14 while demonstrating significant correlations with other items (Appendix Table A4).

## 5 Discussion

The economic burden on cancer patients is a complex problem and a serious one that requires immediate attention and action [33, 34]. In this cross‐sectional study, we investigated the current status and influencing factors of FT in PC patients and explored the relationship between FT and quality of life, positive and negative affect, and social support. We found that PC patients reported a higher FT score of 54.27 ± 14.50 compared to the findings of Arenare et al. [27], who reported a score of 36.5 ± 24.9 in their study of FT levels among 221 cancer patients who were receiving or preparing for treatment at 10 Italian centers using PROFFIT. In the analysis of influencing factors, our results indicated that patients with fewer household savings, more total OOP costs, treatment nonadherence, and unemployment suffered from more severe FT; conversely, increasing positive affect and social support could mitigate FT. (Appendix Table A5).

Consistent with previous findings [35, 36], we found that the adverse effects of FT were more likely to be magnified in patients with lower household economic reserves. Patients with low household savings encounter challenges in achieving early disease screening, timely consultations, and regular follow‐ups, which may lead to more severe symptoms and more complications, increasing the treatment cost for patients and aggravating their FT level. In addition, studies have found [37] that when the cost threatens the financial well‐being of patients' families, they often choose to preserve the financial welfare of their loved ones rather than their own health outcomes, which can result in poorer patient outcomes. Household savings are a key determinant of a family's capacity to pay out‐of‐pocket expenses [38]. Typically, in the face of high cancer care costs, patients with low socioeconomic status are more likely to reach the lower limit of their family's affordability. Therefore, economically disadvantaged cancer patients experience greater economic pressure when confronted with the costs of cancer treatment. Furthermore, patients with limited financial resources have a higher likelihood of treatment nonadherence, which may result in cancer recurrence and increased complications, triggering a vicious cycle of weak financial foundation—bad healthcare behaviors—deteriorating health outcomes—reduced income and savings—exacerbated FT [39].

As cancer treatment progresses, the patient's OOP accumulates further, greatly heightening the financial distress of PC patients. Excessive OOP is a major cause of FT [40], which is part of the cancer treatment journey and a considerable obstacle to providing high‐quality care for cancer patients [41]. High OOP can influence patients' choices of treatment [42], for example, patients who cannot afford OOP may opt for lower‐cost treatment options, even though these options are linked to lower survival and more treatment side effects. In order to offset the cost, patients may deviate from prescribed treatments or discontinue them altogether [42, 43]. It is noteworthy that low OOP does not mean low FT, as patients may forego certain treatments at the expense of their own health benefits. Future research should focus more on patient‐reported outcomes driven by both OOP and patients' subjective economic feelings rather than relying solely on cost data.

The results indicated that retirement was a protective factor for FT; unemployed patients often had higher FT, and undergoing a job change after diagnosis was also significantly associated with higher FT. Given the rapid progression and heavy symptom burden of PC, patients require longer hospital stays and frequent follow‐up visits. Meanwhile, the heavy symptom burden restricts patients' ability to manage substantial workloads, forces them to change their work status, and prompts them to adopt strategies to cope with the disease at the cost of decreasing income and sacrificing career development. Thus, the financial losses suffered by patients after diagnosis include not only the direct cost of the treatment itself but also the reduction of personal income [44]. It needs to be emphasized that diseases can have a lasting negative impact on employment, which means that FT may be a long‐term issue for cancer patients [45]. Return‐to‐work programs may effectively prevent or reduce FT in patients [46]. During treatment, we should be alert to the occurrence of work changes and unemployment in patients. Through multidisciplinary care teams, we can formulate plans involving physical, psychological, and vocational aspects and use platforms such as Internet hospitals to encourage patients to return to work so as to effectively prevent the onset of FT.

In this study, positive affect was negatively correlated with increased FT. The reason may be that positive affect helps to improve one's mental health, promote health‐related behaviors, and enhance patients' health self‐management abilities, thereby reducing the occurrence of complications, improving disease prognosis, and alleviating economic burden. Previous research has found [47] that negative affect and financial distress in cancer patients interacted with each other, with more negative patients perceiving more subjective economic burden, even if their current assets were sufficient to cover cancer expenses. We found that positive affect was a protective factor against FT. Patients exhibiting higher levels of positive affect can face their financial bills with a more optimistic attitude, adopt positive coping strategies to handle economic problems encountered during disease treatment, and actively seek all kinds of financial assistance and social support [48], which alleviates financial pressure to a certain extent. Based on the above information, we recommend that greater attention should be paid to the mental health of PC patients and that group communication and psychological counseling services should be provided to alleviate their subjective feelings regarding FT. Moreover, healthcare staff ought to provide information about treatment costs as early as possible to prevent patients from overestimating their economic burden, improve patients' self‐awareness, and enable them to approach the costs of illness with a more positive perspective.

Inadequate family and social support is one of the key factors linked to FT in PC patients. A high level of social support during the treatment process can help patients access more material, financial, and spiritual assistance [49], buffer the impact of negative events on patients, and improve their ability to cope with financial distress. At the same time, high social support can enhance patients' treatment adherence [50], mitigate their symptom distress, improve their quality of life, and reduce FT. Therefore, healthcare staff should focus on the impact of social support on FT and help patients establish a comprehensive support system by means of peer support and improving continuous care service so as to strengthen their psychological resilience and sense of social support to better cope with economic difficulties.

FT correlates with a worse quality of life [13, 33]. Gupta et al. [51] surveyed patients with hepatocellular carcinoma and found that their quality of life score was 130.3 ± 20.3; Keilson et al. [52] showed that the average FACT‐Hep score of patients with cholangiocarcinoma ranged between 115.23 and 120.12, and we had a score of 101.52 ± 15.09 for PC patients, which reflects a worse quality of life. In our study, 55% of patients chose to sacrifice spending on leisure activities and/or essential goods to pay for medical care. Although these adjustments can temporarily ensure the effectiveness of treatment, in the long run, such behaviors may impact their physical and emotional well‐being and thus reduce their quality of life [13]. Additionally, most patients surveyed expressed concerns about their future financial situation, which could be regarded as a chronic adverse event of cancer treatment [33], not only affecting the quality of life of the patients but also that of their entire family [53]. Nursing staff should routinely carry out FT assessments, identify patients with high FT, equip patients and their families with strategies and information to manage FT, minimize the impact of FT on patients and their families, and ultimately safeguard their quality of life.

It is indisputable that the Chinese government has always been committed to providing support for patients. China has established a multilevel medical insurance system [54], supplementing and extending basic medical insurance with serious diseases insurance, commercial health insurance, and so on to improve families' ability to afford the costs of cancer treatment. However, cancer patients often face serious financial distress due to complex medical insurance policies, lack of understanding of the costs of follow‐up treatment, and irrational means of managing personal assets. These challenges could be addressed through interventions such as financial navigation, which is not common in China [55]. Medical staff in China are often faced with heavy clinical workloads and have no time to popularize insurance and economic knowledge to patients. Financial navigators can address this problem by providing patients with a professional financial navigation plan to help alleviate FT [56]. Healthcare systems should focus on the creation of this position and provide more support for patients.

Previous studies have confirmed that FT leads to worse treatment outcomes. Analyzing the FT of cancer patients and understanding their unique financial needs is becoming increasingly important. PROFFIT excels in this aspect, with its unique FT determinants providing valuable insights for developing personalized intervention plans for patients rather than merely identifying high‐risk groups of FT. Also, personalized intervention plans help to reduce resource waste, allowing limited resources to assist more patients in alleviating their financial hardship. According to our findings, positive affect and work status also exerted a significant impact on PC patients' FT. Therefore, we decided to add the items “我有信心支付我全部的医疗费用” (I am confident that I can pay all my medical expenses) and “我认为疾病治疗对我的工作产生了影响” (I believe that the care of my disease has affected my work) into PROFFIT to better identify the determinants of FT in patients. In addition, the correlation between PROFFIT and FACT2 was not significant in our study, suggesting that FACT2 was perhaps not a crucial determinant of FT in PC patients. However, our study focused exclusively on pancreatic cancer patients, and these items had not been validated in patients with other types of cancer, so we decided to retain these items to enhance the general applicability of PROFFIT.

FT is a dynamic and personalized phenomenon [57, 58]. The cancer‐related costs, patients' attitudes toward treatment, and their choices regarding economic sacrifices change throughout their lives. Several studies have found that FT improves slightly with diagnosis and treatment, but the change in FT over time remains largely unexplored [57, 59]. More studies are needed in the future to track the shifts in FT throughout the entire treatment journey. In addition, patients' risk factors for FT at different disease stages change [57], indicating that FT mitigation measures may also change at different stages. Therefore, we recommend that FT levels of cancer patients be assessed regularly to identify those who are slipping into FT in a timely manner and that interventions for patients with high FT levels be adjusted to make them more targeted. Based on the review of previous longitudinal studies of FT [57, 60, 61], we suggest that FT levels in cancer patients should be assessed once every 6 months to 1 year, and the frequency of assessment should be adjusted appropriately considering the characteristics of the disease and the individual situation of the patient when it is implemented.

## 6 Limitations

Several limitations of this study need to be acknowledged. First, the cross‐sectional design could not evaluate the dynamic changes of PROFFIT score and FT determinants with treatment, nor could it establish the causal relationship between FT and potential determinants. Second, this study was a single‐center study, which limited the generalizability of the findings to a broader population. Third, the study relied on self‐reported data, potentially introducing recall bias. Fourth, the involvement of researchers in filling out the questionnaires during the data collection might cause respondent bias, but the researchers received training related to questionnaire surveys before collecting data and were required to use scientific and neutral language to answer patients' questions to avoid influencing the survey results, which mitigated the respondent bias to some extent. Fifth, a newly translated instrument was used for the FT survey in this study, and the difference in assessment instruments made it impossible to compare the results of this study with other FT studies in China. Sixth, we did not assess FT from the caregiver's perspective, which is critical to comprehensively understanding the factors influencing FT. Lastly, the subjects in this study were recruited from hospitals, and they could be assumed to have higher FT, so we may have overestimated the FT level of PC patients; on the other hand, we did not take into account those patients who gave up treatment for financial reasons, and at this level, we again may have underestimated the FT level of PC patients. We look forward to more large‐sample, multiregional studies of FT in PC patients to verify our hypothesis in the future. Throughout our investigation, we noted that patients in critical condition were likely to refuse the survey, resulting in a lack of data for this demographic.

## 7 Conclusions

This study revealed that FT was very prevalent and persistent among PC patients. Patients exhibiting fewer household savings, more total OOP costs, treatment nonadherence, unemployment, diminished positive affect, and lack of social support are observed to experience severe FT. These findings are beneficial for healthcare providers and policymakers to identify and manage patients with high‐risk characteristics of FT, to further optimize and improve cancer‐related health policies, to safeguard the economic security and well‐being of individuals undergoing cancer treatment, and ultimately to promote health equity in the field of cancer treatment.

## Author Contributions

Jing Wang: data curation, investigation, visualization, project administration, writing – original draft, writing – review, and editing. Jialu Cui: data curation and writing – original draft. Xiaoyuan Wang: investigation and project administration. Zhihua Li: supervision and resources. Yang Liu: supervision and resources. Baoxin Shi: conceptualization, methodology, supervision, resources, writing – review, and editing.

## Ethics Statement

This study was performed in line with the Declaration of Helsinki. The Ethics Committees of Tianjin Medical University approved this study (TMUhMEC20240030). Written informed consent was obtained from patients before the study, informing them of their right to freely choose to participate or withdraw from the study. All collected data were kept strictly confidential and used solely for research purposes.

## Conflicts of Interest

The authors declare no conflicts of interest.

## Declaration of Generative AI and AI‐Assisted Technologies in the Writing Process

No AI tools/services were used during the preparation of this work.

## Data Availability

The data that support the findings of this study are available from the corresponding author upon reasonable request.

## Appendix

**TABLE A1:** Univariate analysis of FT‐determinant dimensions (*N* = 296).

Variable	FACT1 (Mean ± SD)	FACT2 (Mean ± SD)	FACT3 (Mean ± SD)	FACT4 (Mean ± SD)
Demographic information				
Sex				
Male	61.11 ± 20.85	11.11 ± 17.28	59.72 ± 39.80	6.70 ± 11.89
Female	58.69 ± 21.20	9.29 ± 17.46	57.62 ± 40.79	5.79 ± 11.58
t/F/Z/K	0.989	0.903	0.449	0.660
*p*	0.323	0.367	0.654	0.510
Age (years)				
≤ 55	57.49 ± 24.36	9.55 ± 15.26	65.36 ± 38.21	5.74 ± 10.07
56 ~ 65	62.54 ± 17.89	9.29 ± 16.51	66.22 ± 38.73	7.47 ± 13.32
> 65	59.22 ± 20.97	12.06 ± 20.07	43.44 ± 39.95	5.32 ± 11.14
t/F/Z/K	1.590	0.443	10.612***	1.007
*p*	0.452	0.801	< 0.001	0.605
Place of residence				
Cities	58.61 ± 21.63	12.98 ± 20.03	38.81 ± 39.63	4.33 ± 9.90
Townships	61.84 ± 21.54	6.14 ± 11.25	77.63 ± 30.82	8.48 ± 13.78
Rural	61.16 ± 20.04	7.95 ± 14.45	79.36 ± 28.95	8.15 ± 12.89
t/F/Z/K	0.635	5.442	68.815***	8.246*
*p*	0.531	0.066	< 0.001	0.016
Educational level				
Middle school and below	59.58 ± 21.26	13.06	69.95 ± 35.08	8.05 ± 12.42
High school/secondary school	62.22 ± 16.99	9.31 ± 16.99	52.78 ± 41.80	5.46 ± 11.52
Junior college	55.56 ± 21.11	21.21 ± 24.75	45.45 ± 42.75	2.36 ± 7.22
Undergraduate and above	55.21 ± 29.64	16.67 ± 22.77	41.67 ± 40.83	6.25 ± 13.44
t/F/Z/K	2.986	11.278*	17.596**	12.613**
*p*	0.394	0.010	0.001	0.006
Marital status				
Single	33.33 ± 30.43	4.17 ± 8.33	79.17 ± 15.96	0
Married	60.39 ± 20.68	10.51 ± 17.68	58.21 ± 40.22	6.26 ± 11.74
Divorced	55.56 ± 13.61	8.33 ± 13.94	44.44 ± 49.07	7.41 ± 13.46
Widowed	61.11 ± 23.57	8.33 ± 15.39	66.67 ± 40.83	7.41 ± 12.64
t/F/Z/K	2.313	0.279	2.346	0.454
*p*	0.076	0.840	0.504	0.715
Living alone				
Yes	55.56 ± 24.91	5.56 ± 13.26	61.90 ± 39.14	6.35 ± 11.95
No	60.30 ± 20.71	10.61 ± 17.60	58.48 ± 40.36	6.26 ± 11.74
t/F/Z/K	−0.998	−1.508	0.375	0.033
*p*	0.319	0.132	0.708	0.975
Number of children				
Childless	51.67 ± 29.87	6.67 ± 11.65	65.00 ± 34.65	7.78 ± 13.91
One	59.80 ± 21.65	11.08 ± 18.22	51.76 ± 41.70	5.56 ± 10.49
Two	59.78 ± 19.64	7.97 ± 14.30	65.22 ± 37.77	5.92 ± 11.47
Three or more	65.28 ± 16.97	14.58 ± 22.69	80.56 ± 28.52	12.04 ± 17.92
t/F/Z/K	2.080	2.006	10.800*	3.128
*p*	0.556	0.571	0.013	0.372
Disease‐related information				
Numbers of hospitalizations				
≤ 5	55.53 ± 20.92	8.89 ± 16.78	60.42 ± 40.14	5.88 ± 11.50
6 ~ 15	69.25 ± 17.33	13.51 ± 18.33	50.86 ± 40.88	6.51 ± 11.78
> 15	72.78 ± 17.22	13.33 ± 18.78	62.22 ± 38.89	8.52 ± 13.27
t/F/Z/K	39.674***	2.144	1.410	0.679
*p*	< 0.001	0.119	0.246	0.508
Time of illness				
< 3 months	51.71 ± 21.29	6.30 ± 13.43	62.73 ± 38.46	6.91 ± 12.67
3 ~ 6 months	61.05 ± 19.41	13.95 ± 20.43	55.81 ± 42.30	4.78 ± 9.87
6 months ~ 1 year	67.50 ± 18.32	13.33 ± 21.36	51.67 ± 39.33	5.00 ± 9.86
1 ~ 2 years	72.38 ± 13.97	13.81 ± 18.74	60.95 ± 40.41	4.44 ± 10.16
> 2 years	73.21 ± 17.18	10.12 ± 15.28	51.79 ± 42.39	11.11 ± 14.50
t/F/Z/K	53.274***	13.512**	2.966	6.696
*p*	< 0.001	0.009	0.563	0.153
Clinical stage				
I	49.82 ± 23.38	10.82 ± 18.24	61.88 ± 39.38	4.73 ± 8.99
II	60.57 ± 17.68	9.68 ± 17.61	57.53 ± 41.38	7.05 ± 13.39
III	66.99 ± 18.10	8.17 ± 13.89	61.11 ± 37.52	7.63 ± 12.27
IV	69.25 ± 17.33	12.07 ± 18.42	53.45 ± 42.32	6.32 ± 12.33
t/F/Z/K	39.041***	0.521	1.641	1.242
*p*	< 0.001	0.668	0.650	0.743
Lymphatic metastases				
Yes	63.80 ± 19.35	9.55 ± 17.01	56.69 ± 40.47	7.08 ± 13.20
No	55.64 ± 22.03	11.03 ± 17.78	61.03 ± 39.96	5.36 ± 9.77
t/F/Z/K	3.282**	−0.730	−0.927	0.115
*p*	0.001	0.466	0.355	0.909
Distant metastases				
Yes	69.25 ± 17.33	12.07 ± 18.42	53.45 ± 42.32	6.32 ± 12.33
No	57.70 ± 21.25	9.80 ± 17.11	60.01 ± 39.68	6.26 ± 11.61
t/F/Z/K	4.037***	0.891	−1.115	0.038
*p*	< 0.001	0.374	0.266	0.969
Type of treatment received				
Surgery	57.55 ± 21.12	10.11 ± 17.53	60.11 ± 39.64	6.51 ± 11.89
Radiotherapy	66.67	0	0	0
Chemotherapy	69.35 ± 17.15	14.55 ± 19.68	55.37 ± 40.64	6.12 ± 11.40
Immunotherapy	75.00 ± 11.79	25.00 ± 35.36	50.00 ± 70.71	5.56 ± 7.86
Target therapy	69.44 ± 6.80	22.22 ± 25.09	63.89 ± 40.02	5.56 ± 13.61
Traditional Chinese medicine	58.33 ± 28.87	29.17 ± 28.46	12.50 ± 15.96	0
t/F/Z/K	31.214***	12.010*	8.480	2.735
*p*	< 0.001	0.035	0.132	0.741
Types of chronic diseases				
0	58.78 ± 22.46	9.01 ± 15.96	63.85 ± 38.71	5.41 ± 10.88
1	61.05 ± 19.57	11.63 ± 18.79	57.56 ± 40.56	7.24 ± 12.84
2	59.78 ± 20.36	9.78 ± 17.77	50.00 ± 42.02	7.73 ± 12.80
≥ 3	65.63 ± 16.63	15.63 ± 20.61	42.71 ± 41.71	4.86 ± 9.91
t/F/Z/K	0.741	3.347	7.304	1.177
*p*	0.864	0.341	0.063	0.758
Financial information				
Work status				
Full‐time	52.12 ± 24.04	10.85 ± 17.49	60.58 ± 40.31	6.00 ± 13.06
Retired	60.13 ± 20.07	11.92 ± 19.82	45.64 ± 42.15	5.38 ± 11.26
Unemployed	64.56 ± 18.91	7.77 ± 13.37	74.11 ± 31.37	7.55 ± 11.46
t/F/Z/K	12.647**	1.265	25.653***	5.004
*p*	0.002	0.531	< 0.001	0.082
Monthly income (CNY)				
≤ 2000	63.62 ± 19.18	7.53 ± 14.23	77.06 ± 30.19	7.89 ± 11.88
2001 ~ 5000	61.16 ± 21.77	8.26 ± 15.68	58.95 ± 40.88	5.97 ± 12.34
5001 ~ 8000	54.35 ± 20.92	11.96 ± 18.81	46.74 ± 39.85	5.56 ± 11.17
8001 ~ 11,000	50.64 ± 22.35	14.74 ± 18.46	25.00 ± 37.19	5.13 ± 10.99
> 11,000	61.67 ± 15.81	40.00 ± 25.09	28.33 ± 33.38	1.11 ± 3.51
t/F/Z/K	12.765*	25.943***	45.322***	6.189
*p*	0.012	< 0.001	< 0.001	0.185
Household savings (CNY)				
≤ 50,000	37.50 ± 25.00	4.17 ± 8.33	75.00 ± 31.91	11.11 ± 15.71
50,001 ~ 100,000	69.44 ± 22.29	5.56 ± 12.98	81.94 ± 29.69	11.11 ± 16.41
100,001 ~ 150,000	64.75 ± 20.44	7.65 ± 13.11	84.15 ± 26.25	9.47 ± 13.73
150,001 ~ 200,000	60.79 ± 20.45	8.51 ± 14.79	63.43 ± 37.32	4.96 ± 10.11
> 200,000	54.58 ± 20.54	16.25 ± 23.12	26.88 ± 35.53	5.14 ± 11.31
t/F/Z/K	19.595**	8.303	76.124***	10.439*
*p*	0.001	0.081	< 0.001	0.034
Type of medical insurance				
Urban workers' medical insurance	57.73 ± 22.05	11.05 ± 18.11	48.90 ± 41.59	4.91 ± 10.29
Urban resident medical insurance	57.64 ± 19.65	15.28 ± 24.04	61.81 ± 39.77	10.19 ± 14.62
New rural cooperative medical insurance	65.72 ± 17.93	6.63 ± 11.45	78.98 ± 29.09	8.21 ± 13.32
Others	44.44 ± 25.46	27.78 ± 34.69	33.33	0
t/F/Z/K	11.197*	4.358	31.493***	10.766*
*p*	0.011	0.225	< 0.001	0.013
Total OOP costs (CNY)				
≤ 20,000	44.44 ± 17.21	0	47.22 ± 37.14	7.41 ± 13.46
20,001 ~ 50,000	52.86 ± 19.12	10.95 ± 17.73	63.02 ± 39.36	5.71 ± 10.58
50,001 ~ 100,000	60.79 ± 21.68	7.46 ± 15.67	55.40 ± 40.39	5.71 ± 11.85
> 100,000	69.37 ± 18.83	13.75 ± 18.89	58.33 ± 41.42	7.64 ± 12.94
t/F/Z/K	41.483***	12.718**	1.982	0.917
*p*	< 0.001	0.005	0.576	0.821
Knowledge of OOP costs				
Very well	64.07 ± 18.79	9.26 ± 16.55	54.72 ± 40.72	6.17 ± 11.88
Somewhat	55.79 ± 21.44	12.28 ± 19.48	67.89 ± 37.84	5.85 ± 11.09
Hardly at all	41.67 ± 25.07	9.17 ± 13.76	52.50 ± 41.98	7.78 ± 12.54
Not at all	83.33	16.67	33.33	33.33
t/F/Z/K	23.579***	3.415	7.034	4.135
*p*	< 0.001	0.332	0.071	0.247
Impact of disease on work				
No impact	60.24 ± 19.27	10.16 ± 17.79	56.35 ± 40.31	6.38 ± 11.89
Yes impact, overtime/part‐time job/delaying retirement age	66.67	16.67	100	0
Yes impact, early retirement age	77.78 ± 11.79	12.96 ± 20.03	83.33 ± 33.33	8.64 ± 14.46
Yes impact, unemployment/resignation at home	63.89 ± 27.57	6.48 ± 10.13	86.11 ± 25.08	9.88 ± 11.99
Yes impact, taking leave/closing the business	53.33 ± 25.53	11.48 ± 17.34	53.70 ± 40.96	3.95 ± 10.09
t/F/Z/K	14.317**	1.870	14.878**	7.383
*p*	0.006	0.760	0.005	0.117
Treatment nonadherence				
Yes	67.62 ± 19.08	9.96 ± 14.48	68.97 ± 38.22	8.05 ± 13.73
No	56.78 ± 21.01	10.37 ± 18.46	54.47 ± 40.35	5.53 ± 10.74
t/F/Z/K	4.498***	0.970	2.851**	1.272
*p*	< 0.001	0.332	0.004	0.203

*Note:* * Means *p* < 0.05, ** means *p* < 0.01, and *** means *p* < 0.001.

**TABLE A2:** Correlation between FT‐determinant dimensions, quality of life, positive and negative affects, and social support (*n* = 296).

Variable	Correlation with financial toxicity							
	FACT1		FACT2		FACT3		FACT4	
	*r*	*p*	*r*	*p*	*r*	*p*	*r*	*p*
Quality of life	−0.402	< 0.001	−0.031	0.590	−0.145	0.012	−0.157	0.007
Physical well‐being	−0.262	< 0.001	−0.200	0.001	0.012	0.837	−0.078	0.182
Social/family well‐being	−0.196	0.001	0.033	0.574	−0.292	< 0.001	−0.189	0.001
Emotional well‐being	−0.441	< 0.001	0.001	0.997	−0.159	0.006	−0.132	0.024
Functional well‐being	−0.332	< 0.001	0.001	0.998	−0.206	< 0.001	0.217	< 0.001
Specific issues well‐being	−0.369	< 0.001	−0.038	0.510	−0.054	0.351	−0.062	0.286
Positive affect	−0.276	< 0.001	0031	0.593	−0.188	0.001	−0.181	0.002
Negative affect	0.252	< 0.001	0.029	0.618	0.165	0.004	0.177	0.002
Social support	−0.310	< 0.001	0.089	0.129	−0.250	< 0.001	−0.153	0.008

**TABLE A3:** Independent variable assignment.

Independent variable	Assignment method
Sex	Male = 1; female = 2
Age (years)	≤ 55 = 1; 56 ~ 65 = 2; > 65 = 3
Place of residence	Cities = 1; townships = 2; rural = 3
Educational level	Middle school and below = 1; high school/secondary school = 2; junior college = 3; undergraduate and above = 4
Number of children	Childless = 1; one = 2; two = 3; three or more = 4
Numbers of hospitalizations	≤ 5 = 1; 6 ~ 15 = 2; > 15 = 3
Duration of illness	< 3 months = 1; 3 ~ 6 months = 2; 6 months~1 year = 3; 1 ~ 2 years = 4; > 2 years = 5
Clinical stage	I = 1; II = 2; III = 3; IV = 4
Lymphatic metastases	No = 0; yes = 1
Distant metastases	No = 0; yes = 1
Type of treatment received	Surgery (Z1 = 0, Z2 = 0, Z3 = 0, Z4 = 0, Z5 = 0); radiotherapy (Z1 = 1, Z2 = 0, Z3 = 0, Z4 = 0, Z5 = 0); chemotherapy (Z1 = 0, Z2 = 1, Z3 = 0, Z4 = 0, Z5 = 0); immunotherapy (Z1 = 0, Z2 = 0, Z3 = 1, Z4 = 0, Z5 = 0); target therapy (Z1 = 0, Z2 = 0, Z3 = 0, Z4 = 1, Z5 = 0); traditional Chinese medicine (Z1 = 0, Z2 = 0, Z3 = 0, Z4 = 0, Z5 = 1)
Number of chronic diseases	0 = 0; 1 = 1; 2 = 2; ≥ 3 = 3
Work status	Unemployed (Z1 = 0, Z2 = 0); full‐time (Z1 = 1, Z2 = 0); retired (Z1 = 0, Z2 = 1)
Monthly income (CNY)	≤ 2000 = 1; 2001 ~ 5000 = 2; 5001 ~ 8000 = 3; 8001 ~ 11,000 = 4; > 11,000 = 5
Household savings (CNY)	≤ 50,000 = 1; 50,001 ~ 100,000 = 2; 100,001 ~ 150,000 = 3; 150,001 ~ 200,000 = 4; > 200,000 = 5
Type of medical insurance	Urban workers' medical insurance (Z1 = 0, Z2 = 0, Z3 = 0); urban resident medical insurance (Z1 = 1, Z2 = 0, Z3 = 0); new rural cooperative medical insurance (Z1 = 0, Z2 = 1, Z3 = 0); others (Z1 = 0, Z2 = 0, Z3 = 1)
Total OOP costs (CNY)	≤ 20,000 = 1; 20,001 ~ 50,000 = 2; 50,001 ~ 100,000 = 3; > 100,000 = 4
Knowledge of OOP costs	Very well = 1; somewhat = 2; hardly at all = 3; not at all = 4
Impact of disease on work	No impact (Z1 = 0, Z2 = 0, Z3 = 0, Z4 = 0); yes impact, overtime/part‐time job/delaying retirement age (Z1 = 1, Z2 = 0, Z3 = 0, Z4 = 0); yes impact, early retirement age (Z1 = 0, Z2 = 1, Z3 = 0, Z4 = 0); yes impact, unemployment/resignation at home (Z1 = 0, Z2 = 0, Z3 = 1, Z4 = 0); yes impact, taking leave/closing the business (Z1 = 0, Z2 = 0, Z3 = 0, Z4 = 1)
Treatment nonadherence	No = 0; yes = 1

**TABLE A4:** Correlation of PROFFIT score and FT determinants (*N* = 296).

FT determinants	Mean ± SD	Correlation with PROFFIT score	
		*r*	*p*
FACT1	59.97 ± 21.02	0.449	< 0.001
Item 8	42.34 ± 18.85	0.385	< 0.001
Item 9	77.59 ± 32.08	0.381	< 0.001
FACT2	10.25 ± 17.36	0.053	0.363
Item 10	14.08 ± 23.63	0.074	0.205
Item 11	6.42 ± 16.69	0.050	0.392
FACT3	58.73 ± 40.22	0.369	< 0.001
Item 12	58.00 ± 40.57	0.351	< 0.001
Item 13	59.46 ± 41.27	0.374	< 0.001
FACT4	6.27 ± 11.73	0.185	0.001
Item 14	6.87 ± 14.05	0.105	0.070
Item 15	6.76 ± 13.97	0.183	0.002
Item 16	5.18 ± 12.40	0.175	0.002

**TABLE A5:** Patient‐reported outcome for fighting financial toxicity (PROFFIT).

	I do not agree at all	I agree partially	I agree substantially	I very much agree
1. I can afford my monthly expenses without difficulty (e.g., food, rent, transport…).	1	2	3	4
2. My illness has reduced my financial resources.	1	2	3	4
3. I am concerned by the financial problems I may have in the future due to my illness.	1	2	3	4
4. My financial situation affects the possibility of receiving medical care.	1	2	3	4
5. I have reduced my spending on leisure activities such as holidays, restaurants or entertainment in order to cope with expenses related to my illness.	1	2	3	4
6. I have reduced my spending on essential goods (e.g., food) in order to cope with expenses related to my illness.	1	2	3	4
7. I am worried that I will not be able to work due to my illness.	1	2	3	4
8. The National Health Service covers all health costs related to my illness.	1	2	3	4
9. I have paid for one or more private medical examinations for my illness.	1	2	3	4
10. I have paid for additional medicines or supplements related to my illness.	1	2	3	4
11. I have to pay for additional treatment myself (e.g., physiotherapy, psychotherapy, dental care).	1	2	3	4
12. The treatment center is a long way from where I live.	1	2	3	4
13. I have spent a considerable amount of money on travel for treatment.	1	2	3	4
14. Medical staff (i.e., doctors, nurses, etc.) have been helpful throughout my medical care.	1	2	3	4
15. Staff in hospital administration (i.e., for booking appointments, secretaries, etc.) have been helpful throughout my medical care.	1	2	3	4
16. Healthcare staff communicated well with each other in relation to my care.	1	2	3	4
17. I am confident that I can pay all my medical expenses.	1	2	3	4
18. I believe that the care of my disease has affected my work.	1	2	3	4

*Note:* Please rate the following statements according to how strongly you agree with each topic. 1 Means “I do not agree at all”, 2 means “I agree partially”, 3 means “I agree substantially”, and 4 means “I very much agree”. Please circle the options based on your most honest thoughts.
